# Exploring the translational impact of type 1 diabetes on cerebral neurovascular function through ECoG-LSCI

**DOI:** 10.1063/5.0193267

**Published:** 2024-08-08

**Authors:** Shaoyu Yen, Yuhling Wang, Lun-De Liao

**Affiliations:** Institute of Biomedical Engineering and Nanomedicine, National Health Research Institutes, 35, Keyan Road, Zhunan, Miaoli County 35053, Taiwan

## Abstract

Type 1 diabetes mellitus (T1DM) can result in complications such as retinopathy, nephropathy, and peripheral neuropathy, which can lead to brain dysfunction. In this study, we investigated the effects of T1DM on cerebral neurovascular function in mice. Streptozotocin (STZ) is known to induce T1DM in animals; thus, we used an STZ-induced diabetes model to evaluate the effects of hyperglycemia on brain morphology and neurovascular tissue. Neurovascular coupling is the connection between neuronal activity and cerebral blood flow that maintains brain function. The ECoG-LSCI technique combines electrocorticography (ECoG) and laser speckle contrast imaging (LSCI) to detect cortical spreading depression (CSD) as a marker of neurovascular coupling and measure corresponding neurovascular function. Our results suggested that in the STZ group, hyperglycemia affected excitatory neurotransmission and metabolism, leading to reductions in intercellular signaling, somatosensory evoked potential (SSEP) amplitudes, and CSD transmission rates. Western blot data further revealed that brain-derived neurotrophic factor (BDNF) and neuronal nuclear antigen levels were reduced in the STZ group. Abnormalities in glucose metabolism in the brain and increased phosphorylation of AKT and GSK3 are hypothesized to be responsible for these decreases. Overall, this study highlights the importance of glucose metabolism in normal brain physiology and demonstrates that hyperglycemia disrupts neurovascular coupling and affects cerebral neurovascular function and that the degree of CSD is positively correlated with the extent of brain tissue damage. Further research is essential to gain a complete understanding of the related mechanisms and the implications of these findings.

## I. INTRODUCTION

Type 1 diabetes mellitus (T1DM) is a condition that typically results from an autoimmune response. The destruction of pancreatic β cells is caused by high levels of proinflammatory cytokines produced by macrophages and T cells of the immune system. This destruction results in decreased insulin secretion and thus hyperglycemia. Many clinical studies have shown that diabetes patients are prone to complications, such as cardiovascular disease, kidney disease, diabetic retinopathy, cognitive impairment, and neurodegenerative diseases.^1–4^ T1DM can be modeled by the administration of streptozotocin (STZ) to study diabetic glucotoxicity in β cells. STZ selectively accumulates in pancreatic β cells through glucose transporter 2 (GLUT2). GLUT2 enters β cells and partially destroys the pancreas. A decrease in the mass of β cells ultimately results in their death.^5^ STZ recognizes GLUT2 receptors, which are abundantly expressed on the plasma membrane of β cells. Therefore, pancreatic β cells are a specific target of STZ. Because GLUT2 is also found in small amounts in the liver and kidneys, large doses of STZ can also damage liver and kidney function. After ingestion, STZ is rapidly metabolized in the liver and rapidly eliminated via renal excretion; therefore, STZ is indeed transient (its half-life in the serum after intravenous injection is 15 min^6^) its response to sustained hyperglycemia is detrimental to the liver, and it exhibits negligible acute renal toxicity.^7^ Any further functional impairment of the liver and kidneys following STZ excretion may be attributed to the effects of diabetic hyperglycemia. In addition to affecting pancreatic β cells, STZ also systematically damages organs that express GLUT2, such as the kidneys and liver, but it does not directly affect the brain because the blood–brain barrier lacks this transporter. This information constitutes the basis for studying the mechanisms of STZ-induced diabetes complications in the pancreas, the liver, the kidneys, and other organs, such as the brain.^8^

Hyperglycemia is a condition characterized by elevated concentrations of glucose in the circulating blood, typically exceeding 250 mg/dL. An inability to maintain normal blood sugar levels in the body due to inadequate insulin production or insulin resistance can lead to a metabolic disorder known as diabetes. This condition causes glucose to accumulate in the bloodstream instead of being transported to cells. Hyperglycemia has been associated with several complications, including neuropathy, retinopathy, poor wound healing, and both microvascular and macrovascular diseases.^9^ The brain uses glucose as its main source of energy, and glucose consumption is closely linked to neuronal activity. Therefore, the proper functioning of neurons is highly reliant on a consistent and adequate supply of glucose.^10^ Arterioles are constricted or dilated as needed to regulate local blood flow. This regulatory mechanism helps maintain a steady supply of glucose to neurons, which is critical for the survival and normal function of these cells.

According to the literature, protein kinase B (AKT) and glycogen synthase kinase 3 (GSK3) are sensitive to glucose, and hyperglycemia increases the phosphorylation of AKT and GSK3 in the brain.^11^ Insulin receptors in nearly all vertebrate tissues, including the brain, are associated with essential signaling pathways, including the AKT and GSK3 signaling pathways. AKT has been shown to regulate the expression of proteins involved in the neuronal function. Dysregulation of the phosphoinositide 3-kinase/protein kinase B (PI3K/AKT) pathway is associated with many human diseases, including cancer, diabetes, and cardiovascular and neurological disorders.^12^ In insulin-responsive tissues, insulin signaling activates AKT, thereby inactivating GSK3. AKT regulates the storage of glucose in the form of glycogen by phosphorylating GSK-3 at N-terminal serine residues (GSK-3α Ser21 and GSK-3β Ser9), thereby inhibiting its kinase activity. GSK-3β is a key enzyme in glycogen synthesis and plays an important role in regulating blood sugar levels.^13^ The enzyme GSK3 plays a crucial role in regulating glycogen synthesis in the body. Specifically, GSK3 phosphorylates and inhibits glycogen synthase, which is necessary for the synthesis of glycogen. However, the activity of GSK3 can be inhibited by the enzyme AKT, and this inhibition leads to the dephosphorylation and activation of glycogen synthase, which increases the rate of glycogen synthesis. According to the literature, GSK-3β is a critical factor that contributes to both insulin deficiency and insulin resistance.^14^ GSK-3β also participates in diverse cellular processes, including but not limited to glycogen metabolism, insulin signaling, cell proliferation, neuronal function, tumorigenesis, and embryonic development.

Neuropathy is one of the main complications leading to diabetes, and deficiency of neurotrophic factors is considered one of the important causes of neuropathy.^15^ Brain-derived neurotrophic factor (BDNF) is widely distributed throughout the central nervous system, including the hippocampus, cortex, hypothalamus, brainstem, and spinal cord.^16^ This factor plays crucial roles in promoting neuronal survival and growth, synaptic plasticity, neurogenesis, neural differentiation, hypoglycemia, neurotoxicity, and cerebral ischemia, which makes it an important factor in neuroprotection.^17^

Diabetes is associated with complications related to macrovascular damage, including macrovascular diseases such as coronary artery disease, peripheral vascular disease, and stroke.^18^ Neurovascular function involves regional cerebral blood flow (rCBF) changes driven by neural activity in the same region. The detection of changes in neuronal activity through the measurement of local hemodynamics is possible because of neurovascular activity. Techniques, such as functional magnetic resonance imaging (fMRI) and laser speckle contrast imaging (LSCI), allow the analysis of local hyperemia and the detection of changes in cerebral blood flow (CBF) or blood oxygenation, making them reliable methods for mapping changes in neuronal activity.^19^ LSCI has proven to be a feasible method for detecting variations in cortical CBF in various animal models. This noninvasive imaging technique may offer valuable insights into the intricate neural mechanisms in these models, which have not yet been elucidated. Thus, the use of LSCI in animal studies is a promising approach for addressing fundamental unanswered questions in the field of neuroscience.^20^ In this study, we examined the CBF velocity via LSCI, which has a relatively good resolution.

Cortical spreading depression (CSD) was initially observed by Leão *et al.* Electroencephalographic (EEG) recordings of anesthetized rabbits revealed that strong electrical stimulation of a single point on the cortical surface resulted in a reversible reduction in the amplitude of cortical waves or their suppression, although complete recovery after a period was observed.^21^ Cortical stimulation initiates the reversible propagation of CSD, which spreads in all directions to distant cortical areas. This process is accompanied by vasodilation of the cortical vessels.^22^ Furthermore, cortical tissue exhibits gradual changes in the electrical potential through direct current (DC), which is indicative of a slow all-or-nothing DC signal, a characteristic hallmark of CSD.^23,24^ CSD propagates at approximately 2–5 mm/min in cortical tissue, whereas electrical signals travel much faster in neurons, on the order of tens of meters per second.^25^ CSD is a phenomenon characterized by transient and diffuse depolarization of neurons, and this depolarization can propagate like a wave across the surface of the cortex. CSD can affect normal brain tissue and induce changes in CBF, glucose metabolism, and transmembrane ion transport. These changes, however, are reversible. To simultaneously examine neurovascular function in target brain regions, electrocorticography (ECoG) is an ideal method for acquiring real-time neural signals from specific large brain regions to assess changes in somatosensory evoked potentials (SSEPs). In recent years, advanced optical imaging techniques have been employed to evaluate CBF after stroke. Diffuse optical imaging (DOI) and LSCI are among the cutting-edge techniques used for this purpose.^26^ DOI, for example, is well known for its ability to measure blood flow and oxygenation.^27^

Many studies have shown that diabetes affects the brain and reduces blood sugar metabolism in the brain, causing abnormalities. In this study, we utilized ECoG integrated with LSCI (hereinafter referred to as ECoG-LSCI) to comprehensively assess neurovascular function. The use of ECoG to record the response of the forepaw to electrical stimulation allows changes in blood flow to be observed, and 4 M KCl is used to induce CSD as an indicator of neurovascular responses in the brain. LSCI can be used to reliably assess CBF changes after CSD to evaluate alterations in brain function. ECoG-LSCI can be used for simultaneous observation of neurovascular responses. Through western blotting, we explored the cellular and molecular mechanisms underlying the alterations in the levels of neurotrophic factors and insulin-regulated proteins in the brains of mice in both the control and STZ groups. Our goal was to identify the underlying changes that contribute to alterations in brain function.

## II. RESULTS

### A. Changes in the body weights and blood glucose levels of STZ-induced diabetes model mice

**FIG. 1.**
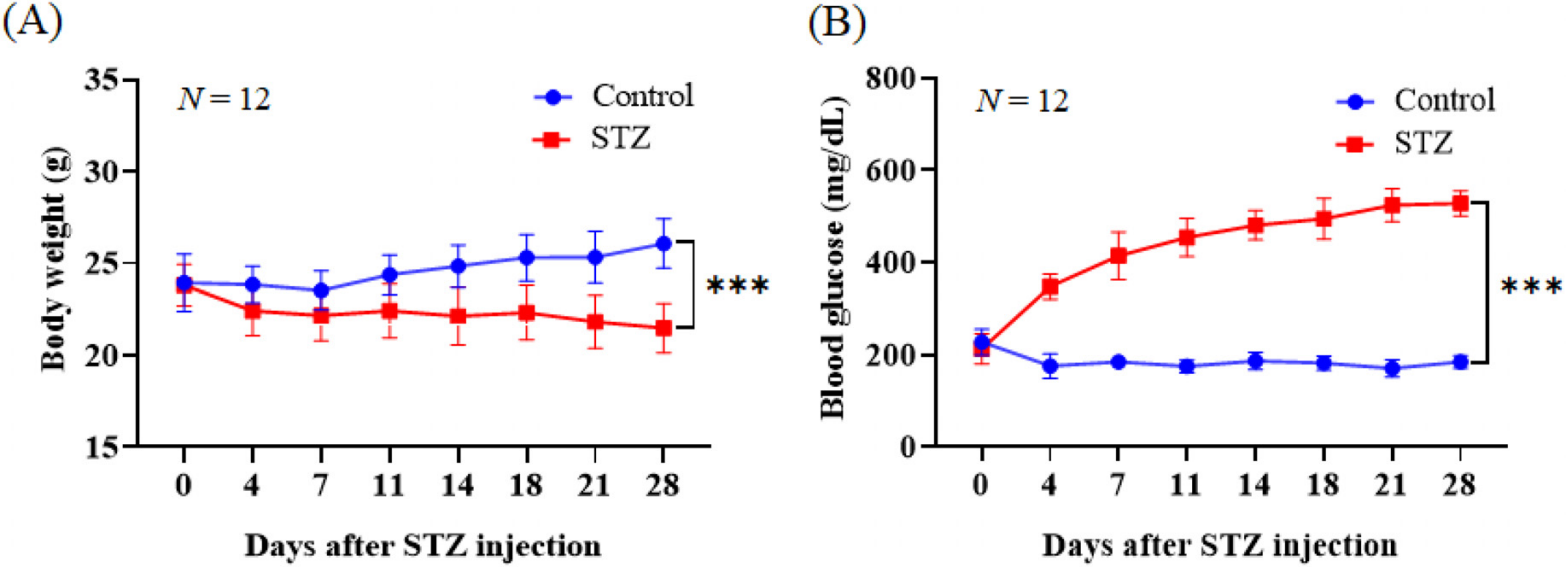
Changes in the blood glucose levels and body weights of STZ-induced diabetes model mice. The mice in the STZ group were intraperitoneally injected with STZ (55 mg/kg/d) for five consecutive days, whereas the control mice received citrate buffer. (a) Body weight changes in the control and STZ groups. Control group vs STZ group, *** *p* < 0.001. (b) Blood glucose concentrations increased several weeks after STZ injection. Control group vs STZ group, *** *p* < 0.001. *n* = 12 per group.

Blood glucose levels were measured 1 week after the mice had adapted to the environment. The average nonfasting blood glucose concentration of this cohort of mice was 220.12 mg/dl, and the mice were randomly divided into a control group and a diabetes group. To accurately measure blood sugar levels, the blood glucose machine was first calibrated. A glucose control solution was used for analysis. To confirm the successful establishment of the hyperglycemia animal model, the body weights and blood glucose levels of the mice were measured on days 0, 4, 7, 11, 14, 18, 21, and 28 after STZ injection. Over time, the mice in the STZ group lost weight, but those in the control group gained weight, and our data revealed significant weight loss [26.08 vs 21 g, *p* < 0.05; Fig. 1(a)]. The blood glucose levels of the mice in the STZ group were significantly greater than those of the mice in the control group [184.8 vs 527.8 mg/dl, *p* < 0.05; Fig. 1(b)].

### B. Changes in neurovascular function in STZ-induced diabetes model mice as measured by ECoG-LSCI

**FIG. 2.**
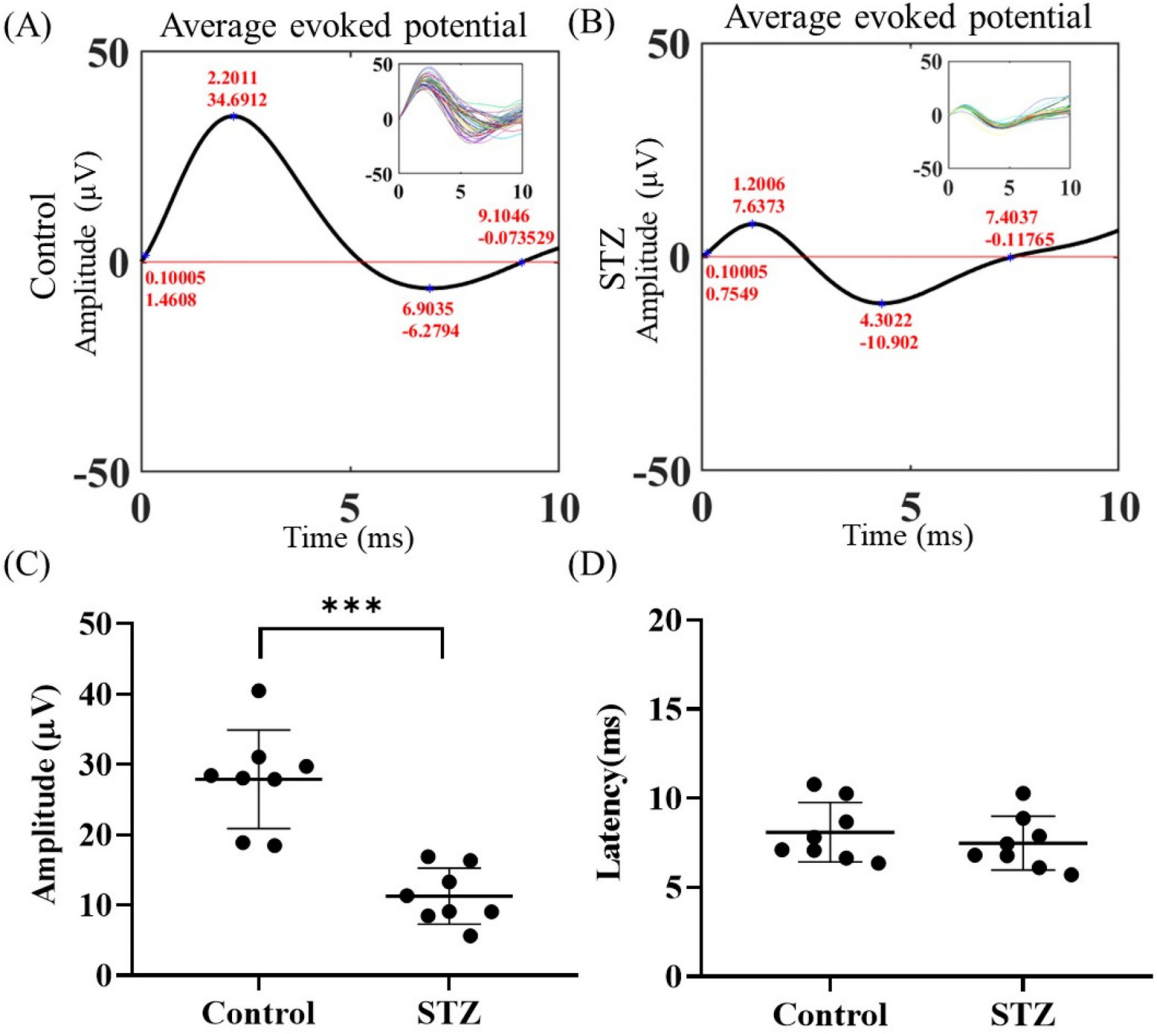
ECoG recordings of control mice and mice with STZ-induced diabetes after electrical stimulation. (a) Mean evoked potentials in the control group. The upper right panel shows the calibrated evoked potentials. (b) Average evoked potentials in the STZ group. The upper right panel shows the calibrated evoked potentials. (c) Differences in evoked potentials between the control and STZ groups. Control group vs STZ group, *** *p* < 0.001. (d) Conduction latency. Control group vs STZ group; *p* = 0.4595; no significant difference. Column analysis of the electrophysiological results was performed via MATLAB and GraphPad Prism (control and STZ groups; *n* = 8 per group), and the differences between the control and STZ groups were analyzed via unpaired *t* tests.

To study the effects of diabetes on brain function, we established a diabetes mouse model. Four weeks after model induction, the mice were subjected to craniotomy for ECoG-LSCI. Figures 2(a) and 2(b) show the average amplitudes of the evoked potentials in the control and STZ groups. In the upper right corner, the SSEP waveform, which includes the P1 amplitude, N1 amplitude, P1 latency, and N1 latency, is displayed. In the control group, the P1 amplitude was 34.69, the N1 amplitude was −6.27, the P1 latency was 0.10, and the N1 latency was 9.10. In the STZ group, the P1 amplitude was 7.63, the N1 amplitude was -10.90, the P1 latency was 0.10, and the N1 latency was 7.40. Comparisons of the SSEP amplitude and latency are shown in Figs. 2(c) and 2(d), respectively. The SSEP amplitude in the STZ group was significantly lower than that in the control group (*p* < 0.05), whereas no significant difference in latency was observed between the control and STZ groups.

**FIG. 3.**
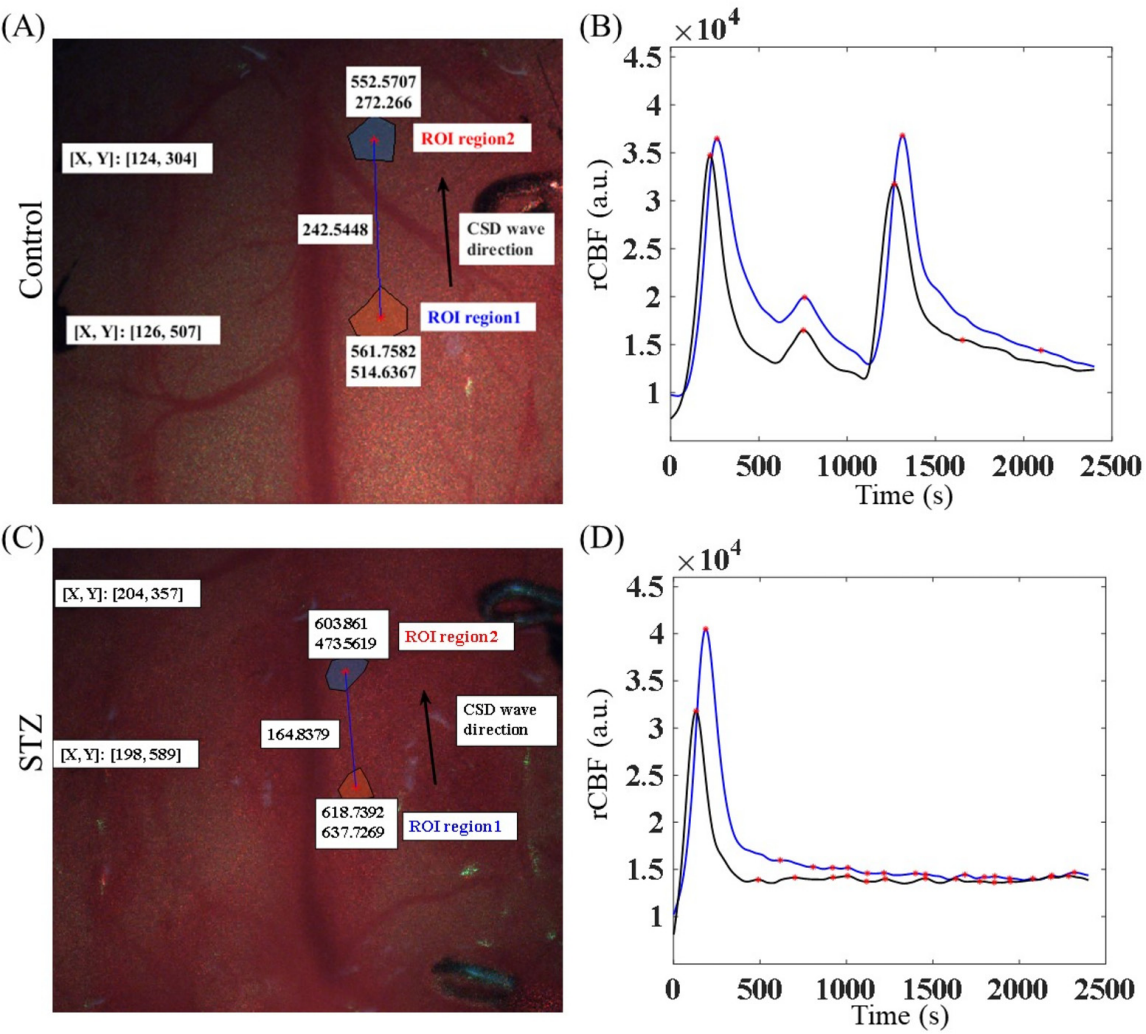
Effects of STZ-induced diabetes on CSD decay time and velocity calculations. (a) The brains of the mice in the control group were photographed via LSCI. (b) CSD over time in the control group. (c) The brains of the mice in the STZ group were also photographed via LSCI. (d) CSD over time in the STZ group. The arrows in panels (a) and (c) indicate the direction of CSD.

Our data revealed that changes in rCBF occurred during KCl-induced CSD in both the control and STZ groups, as depicted in Figs. 3(a) and 3(c). The regions of interest (ROIs) chosen for the rCBF calculations are presented in Figs. 3(b) and 3(d). The CSD velocity of the STZ group (1.17 mm/min) was significantly lower than that of the control group (4.25 mm/min). In addition, we also observed that CSD was not easily induced in the STZ group, possibly because of brain damage.

**FIG. 4.**
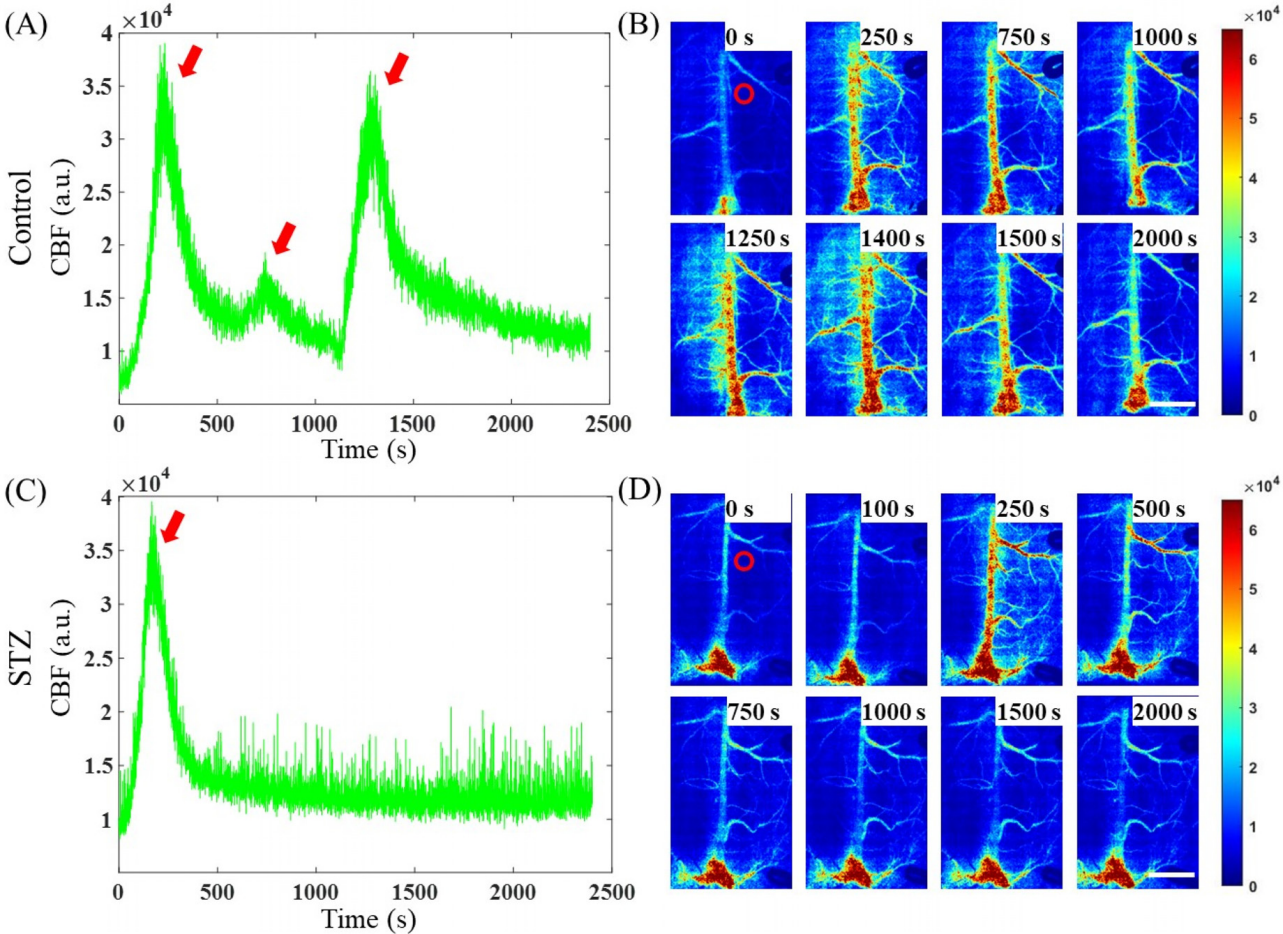
LSCI was used to record CBF during KCl-induced CSD in the control and STZ groups. (a) Analysis of CBF in the control group. The red arrows represent the time points at which CSD was induced, i.e., 250, 750, and 1250 s. The graph corresponds to the figures on the right. (b) Dynamic changes in blood flow in the control group at 0, 250, 750, 1000, 1250, 1400, 1500, and 2000 s. (c) Analysis of CBF in the STZ group. The red arrow represents the CSD at 250 s. The graph corresponds to the figures on the right. (d) Dynamic changes in blood flow in the STZ group at 0, 100, 250, 500, 750, 1000, 1500, and 2000 s. The dark blue areas in the image indicate areas with low blood perfusion, whereas the dark red areas indicate areas with high blood perfusion. The ROIs used are marked by red circles in panels (b) and (d). Videos of the blood flow changes summarized in (b) and (d) are shown in **Supplement 1** and **Supplement 2**, respectively. Scale bar = 2 mm.

**FIG. 5.**
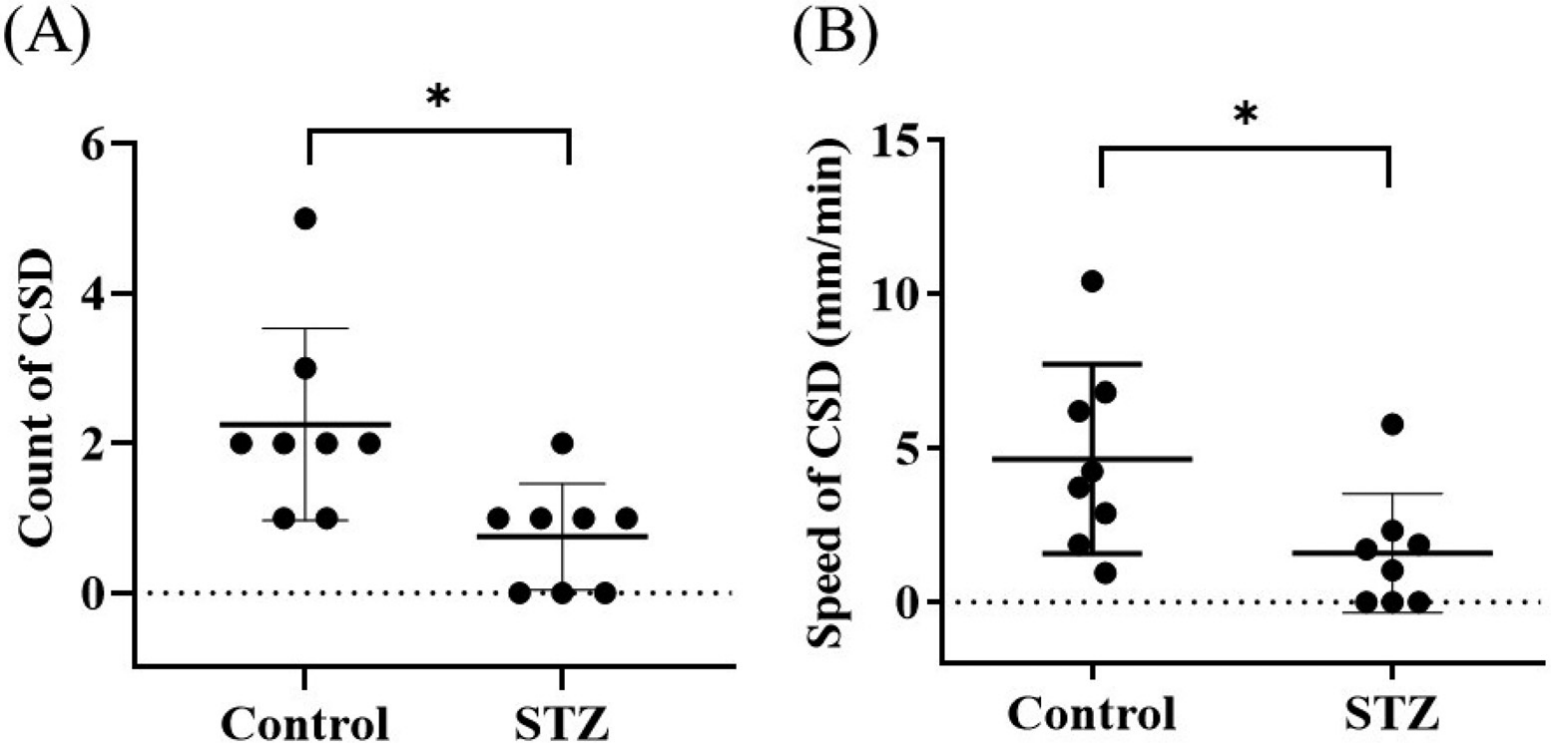
Effects of STZ-induced diabetes on neurovascular coupling in the brain. (a) Number of CSD-related events. Control group vs STZ group, * *p* < 0.05. (b) CSD speed in the control and STZ groups. Control group vs STZ group, * *p* < 0.05. Unpaired *t* tests were conducted to analyze the differences between the control and STZ groups (*n* = 8 in each group).

Figures 4(a) and 4(c) show representative images of the rCBF changes during KCl-induced CSD in the control and STZ groups prior to smoothing of the velocity curve. The images in Figs. 4(b) and 4(d) (**Supplement 1** and **Supplement 2**) show the corresponding rCBF distributions calculated from speckle images during CSD, with the red circles representing the ROIs for measuring blood flow changes. Our results revealed three peaks during CSD in the control group but only 1 peak in the STZ group, and in most of the mice in the STZ groups, blood flow did not return to baseline levels after CSD induction. The statistical analysis revealed that the number of CSD events in the STZ group was significantly lower than that in the control group [Fig. 5(a); *p* < 0.05]. Furthermore, the CSD speed of the STZ group was also significantly lower than that of the control group [Fig. 5(b); *p* < 0.05].

**FIG. 6.**
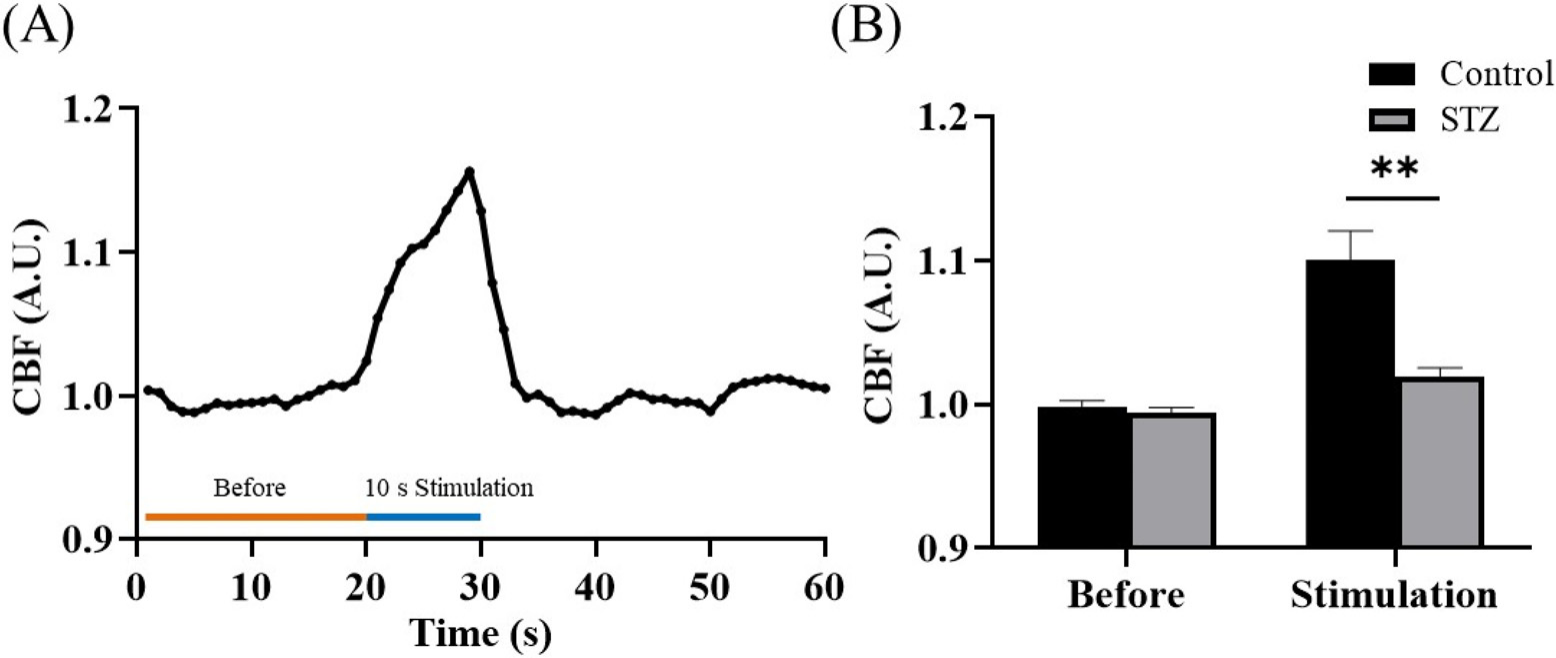
Changes in CBF in STZ-induced diabetes model mice after electrical stimulation. (a) Changes in blood flow in the mouse brain were recorded for a total of 60 s before, during, and after electrical stimulation. At 20 s, the mice were electrically stimulated for 10 s. (b) No significant difference was found between the prestimulation control and STZ groups. Control group vs STZ group, *p* = 0.4595. Cerebral perfusion was significantly reduced during stimulation in the STZ group. Control group vs STZ group, ** *p* < 0.01.

We also observed changes in CBF under electrical stimulation. First, the baseline value was recorded for 20 s, and electrical stimulation was then applied for 10 s beginning at the 20th second. The blood flow velocity changed significantly during electrical stimulation [Fig. 6(a)]. No significant difference in blood flow was found between the control and STZ groups before stimulation, and after electrical stimulation, blood perfusion in the control group was significantly greater than that in the STZ group [Fig. 6(b); *p* < 0.05].

### C. Effects of STZ-induced diabetes on the mouse brain

**FIG. 7.**
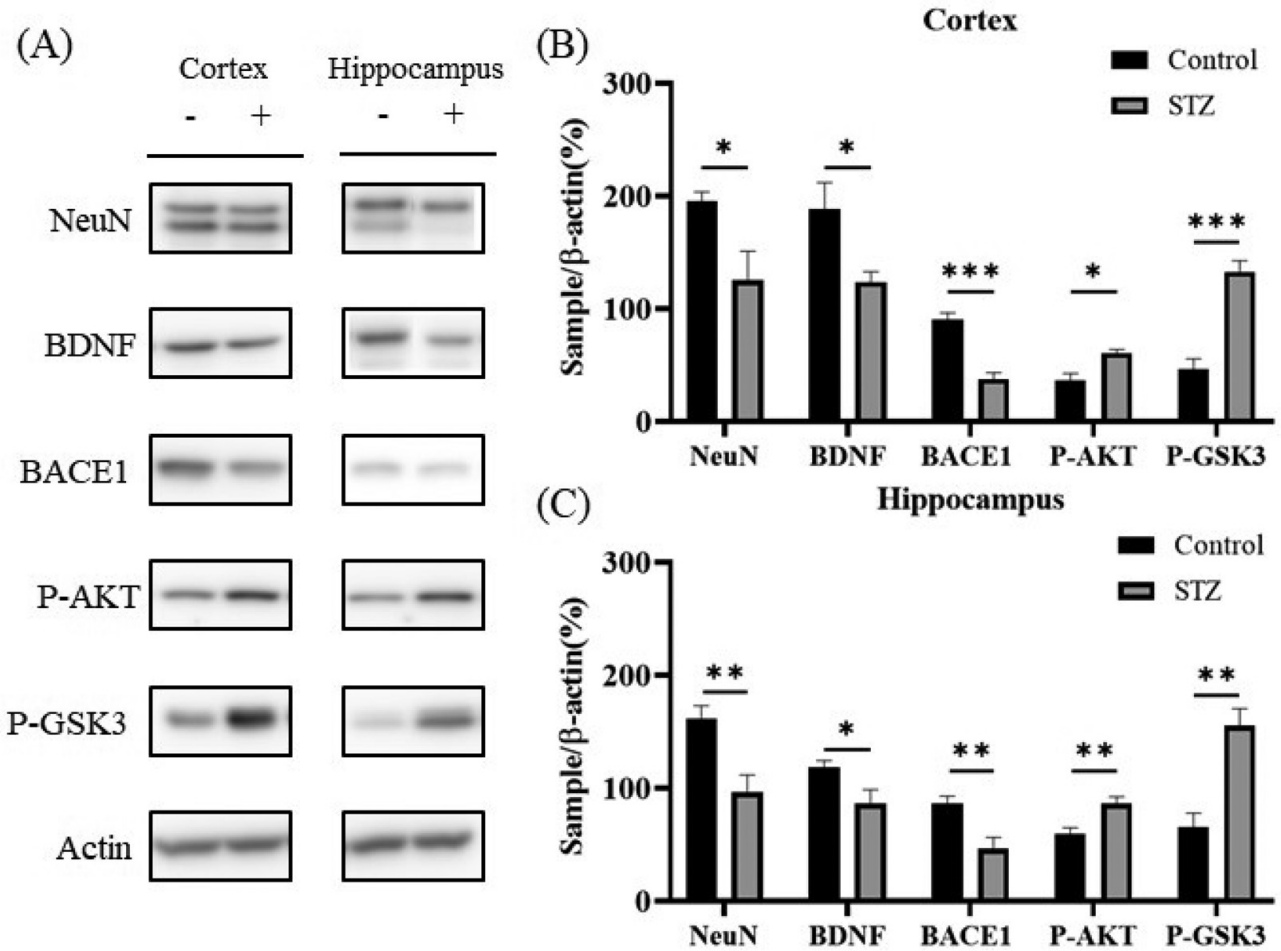
The effects of STZ-induced diabetes on the expression of neurotrophic factors varied in the cortex and hippocampus. (a) Representative western blot images of NeuN, BDNF, BACE1, P-AKT, P-GSK3, and β-actin (*n* = 6 in each group). (b)–(c) Quantification of NeuN, BDNF, BACE1, P-AKT, P-GSK3, and β-actin levels. β-Actin was used as an internal control. In the western blot images, “−” indicates the control group, and “+” indicates the STZ group. Multiple *t* tests, one per row; *** *p* < 0.001, ** *p* < 0.01, and * *p* < 0.05.

To analyze the effects of STZ-induced diabetes on protein expression in the brain, we sacrificed the mice, collected their brains, isolated the cortex and hippocampus for western blotting [Fig. 7(a)], and then performed quantitative analysis via ImageJ. Our data revealed that BDNF expression was significantly reduced in the cortex and hippocampus in the STZ group, that neuronal nuclear antigen (NeuN) expression was significantly reduced in the cortex and hippocampus in the STZ group, and that BACE1 expression was significantly reduced in the cortex and hippocampus in the STZ group [Figs. 7(b) and 7(c); *p* < 0.05].

In the STZ group, hyperglycemia caused glucose imbalance and altered AKT and GSK3 phosphorylation in the brain. Our western blot analysis revealed significant increases in P-AKT (S473) levels in the cortex and hippocampus. P-GSK3 levels were significantly increased in the cortex and hippocampus [Figs. 7(b) and 7(c); *p* < 0.05]. Insulin-producing β cells rapidly degenerated in the pancreas of mice receiving STZ, resulting in greatly reduced insulin production and elevated blood glucose levels. Taken together, these results indicate that insulin deficiency modulates processes in the mouse brain relevant to STZ-induced diabetes.

## III. DISCUSSION

### A. Effects of STZ-induced diabetes on brain function in mice

Cognitive deficits and neurophysiological, neurochemical, and neuroradiological alterations in the brain have been observed in individuals with diabetes, as documented in a 1994 study on brain function in individuals with diabetes mellitus.^30^ Many factors are involved in the pathogenesis of diabetes-associated brain dysfunction, and vascular disease is one of the main factors associated with alterations in the cerebrovascular response and thickening of the capillary basement membrane.^31,32^ Glucose uptake and blood flow are promoted by insulin, indicating that they regulate vascular and metabolic processes.^33^ In the brain, diabetes progressively damages the vascular endothelium, making vessel walls thicker, more permeable, and less responsive to endogenous modulation of the vascular tone.^34,35^ Diabetes is a chronic disease caused by insufficient production/secretion of insulin by the pancreas or insulin resistance and is characterized by uncontrolled hyperglycemia and concomitant metabolic disturbances. Although T1DM and type 2 diabetes mellitus (T2DM) are both characterized by hyperglycemia, T2DM is mainly a metabolic disease and may be associated with insulin resistance and other processes in addition to obesity. Compared with control individuals, patients with T1DM do not gain weight, which was also observed in our study.

Diabetes is a complex disease that can affect different parts of the body, including neurons, stellate cells, and cerebral blood vessels. Studies have shown that individuals with diabetes may experience a reduction in neurovascular coupling and cerebrovascular reactivity. This finding suggests that the blood flow in the brain may decrease, which can lead to a reduction in the diameter of the blood vessels. Such changes in the vascular structure and function are often among the early features of diabetes and other related diseases.^36^ The transmission of related information between nerve cells results in defects in synaptic plasticity, and a decrease in the conduction speed of impulses in the brain is reflected by changes in the latency of increased evoked potentials.^37^ Our data revealed no significant difference in evoked potential latency between the control and STZ groups. This may have been because the induction time was only 1 month, which may have been insufficient to produce a significant change in latency. Nonetheless, the evoked potential amplitude of the STZ group was diminished [Figs. 2(c) and 2(d)]. It is speculated that diabetes can impair peripheral nerve function. Vascular hemoglobin, as the main carrier of oxygen, is related to the vascular blood oxygen response. The brain requires sufficient oxygen to maintain its normal function, and diabetes impairs the important links among nutrient supply, CBF, and metabolic demand. If high blood glucose levels persist, glycosylated hemoglobin levels also remain high, affecting the blood oxygen response in blood vessels and damaging blood vessels in the brain that carry oxygen-rich blood. When the brain receives too little blood, brain cells die, leading to the development of vascular dementia. CSD (a self-propagating wave of membrane/tissue depolarization) is associated with marked changes in tissue metabolism and blood flow. CSD can occur in the normal brain after brain injury. Thus, CSD can be associated with a normal state in healthy individuals or a reduction in the response of rCBF (via the upstream blood supply) to energy demands in disease. Hyperglycemia counteracts the effects of increased extracellular potassium concentrations, thereby inhibiting CSD initiation and propagation.^38^ In hyperglycemia, increased energy storage delays disruption of the transmembrane ion gradient, increasing the latency of induced CSD and reducing its propagation velocity. These mechanisms result in decreases in the CBF velocity and fewer CSD events [Figs. 5(a) and 5(b)]. As mentioned above, hyperglycemia impairs blood flow regulation, which is also consistent with the data from our electrical stimulation experiment. Relatedly, the blood flow velocity of the STZ group was significantly slower than that of the control group [Fig. 6(b)].

### B. Effects of brain-related proteins in type 1 diabetes

Diabetes has been well documented to damage the brain. The mammalian brain relies on glucose as the main energy source, and tight regulation of glucose metabolism is crucial for brain physiology.^39^ Brain nerve cells need insulin to absorb sugar. Astrocytes ingest sugar from the blood and transport it to nerve cells, which convert sugar into energy. Hyperglycemia significantly reduces cell viability and induces apoptosis and the loss of hippocampal neurons. BDNF provides nutritional support for neurons, protects the brain from injury, and participates in the regulation of energy and glucose homeostasis by interacting with TrkB receptors.^40^ BDNF regulates glucose metabolism by increasing insulin sensitivity and pancreatic insulin production.^41^ Synaptic plasticity is a crucial neurochemical process that plays a major role in learning and memory.^42^ The alterations in synaptic protein expression observed in diabetes are caused by several factors, one of which is hyperglycemia. Another potential mechanism underlying the weakened neurovascular coupling response in the diabetic brain may be impaired neuronal function. Our results revealed that hyperglycemia decreases BDNF and NeuN expression (Fig. 7), causing cell damage and complications.

**FIG. 8.**
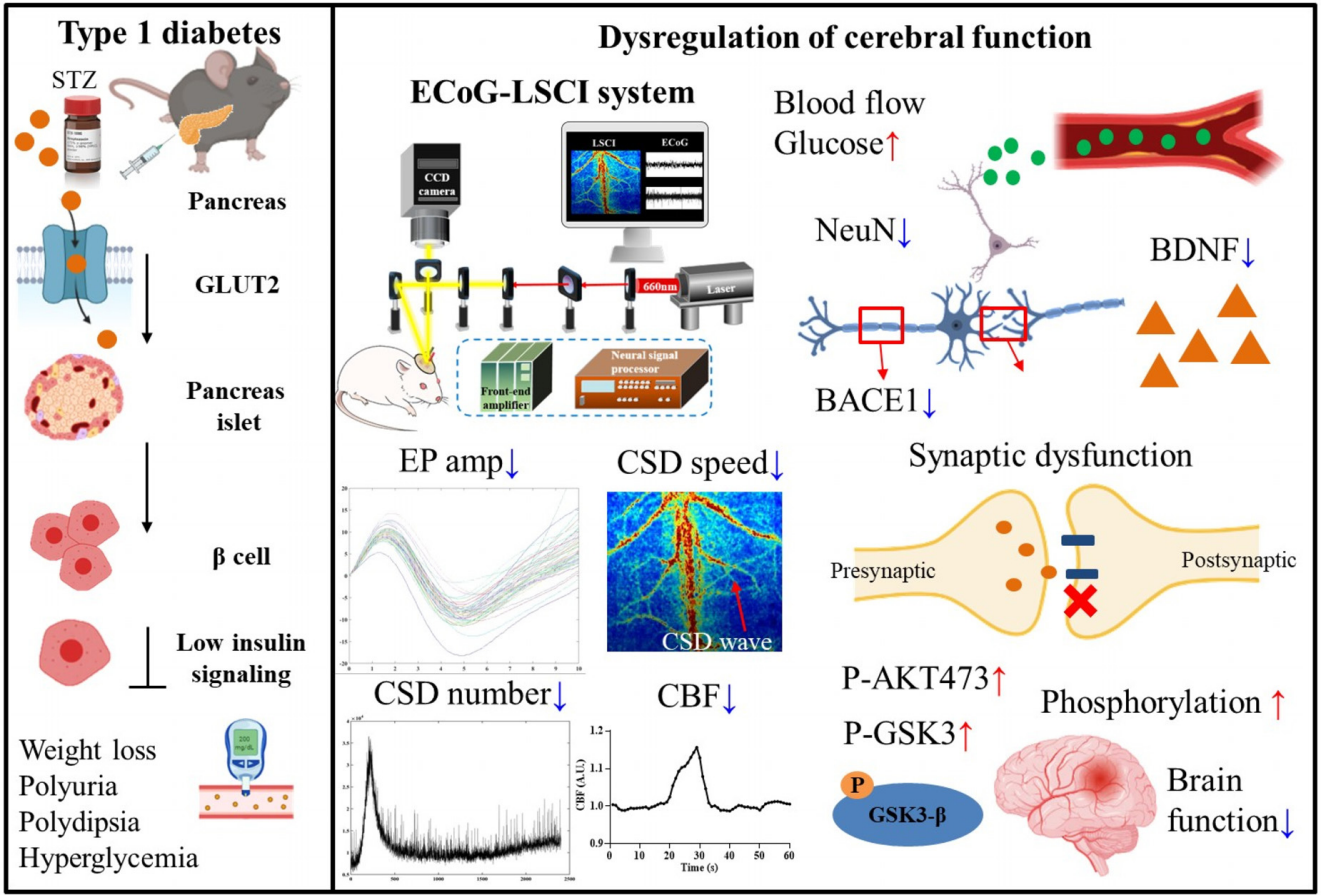
Summary of the effects of STZ-induced diabetes on related brain functions. STZ-induced diabetes is associated with weight loss, polyuria, thirst, hyperglycemia, and impaired brain function. The impairment of physiological neurovascular coupling leads to decreased neuronal activity, an imbalance in ion homeostasis, and abnormal blood flow regulation. Increases in glucose concentrations in the blood lead to tissue damage, resulting in decreases in NeuN, BDNF, and BACE1 levels; synaptic dysfunction; and degeneration of peripheral nerve myelin. Elevated glucose levels lead to the hyperphosphorylation of AKT (S473) and GSK3. NeuN: neuronal nuclear antigen; BDNF: brain-derived neurotrophic factor; BACE1: β-secretase 1; P-AKT (S473): AKT phosphorylated at serine 473; and P-GSK3: phosphorylated glycogen synthase kinase-3.

Diabetes is associated with many complications, and neurological dysfunction often results in sensory deficits that can lead to wound formation or deterioration. Hyperglycemia-induced tissue damage in diabetes patients is usually indirect and caused by blood flow or vascular disturbances. Peripheral nerve demyelination and loss of peripheral function in diabetic patients are partly caused by hyperglycemia, which directly affects Schwann cell apoptosis. According to histopathological studies, rats with STZ-induced diabetes present decreases in the mean myelin content (BACE1 levels) and the myelin/axon ratio, an increased endoneurial space, and reductions in the conduction velocity and pain thresholds. Axonal degeneration is manifested mainly by a decrease in the amplitude of motor or sensory action potentials.^43^ Consistently, our findings revealed that BACE1 expression was significantly lower in the STZ group than in the control group (Fig. 8).

The prevalence of age-related diseases, such as diabetes and Alzheimer's disease (AD), is increasing. People with diabetes are at greater risk of developing AD than healthy people. In addition to AD, it is associated with several metabolic syndromes, including atherosclerosis, hypertension, hyperglycemia, type 2 diabetes, and many other neuropsychiatric disorders, such as depression, Parkinson's disease, and Huntington's disease. Cognitive dysfunction and synaptic damage are strongly associated with T1DM and AD.^44^ Diabetes increases phosphorylation of Akt and GSK3 in the brain, a link between AD and regulation of the GSK3 pathway.^45^ If GSK3 is dysregulated in the brain in AD, it may affect the brain's inflammatory response, causing microglia to continue to secrete neurotoxic inflammatory mediators, resulting in damage to neighboring neurons and contributing to the neurodegenerative process. BDNF levels and function appear to be disrupted by and related to insulin resistance in diabetes. BDNF is a key neurotrophic molecule that has been shown to enhance synaptic plasticity and improve learning and memory in most nerve cells and peripheral systems. Disruption of BDNF expression and function has been found at different stages of AD.^46^ BDNF is associated with AD-related pathology, including neuroinflammation, neuronal apoptosis, and cognitive decline.

## IV. CONCLUSION

Diabetes is associated with numerous complications characterized by electrophysiological, structural, neurochemical, and neurodegenerative changes. Imbalanced blood sugar levels can accelerate the development of neurological complications. Hyperglycemia affects nerve conduction and metabolism, resulting in decreased signaling and BDNF expression. The cellular and molecular mechanisms linking diabetes to vascular dysfunction and cognitive impairment are complex, and disease-associated neuronal/glial degeneration may be a major contributor to behavioral abnormalities. This study provides a reliable assessment of brain function in patients with T1DM and may aid the development of new drug therapies to prevent and treat cerebrovascular dysfunction.

## V. METHODS

### A. Combination of ECoG and LSCI for brain imaging

**FIG. 9.**
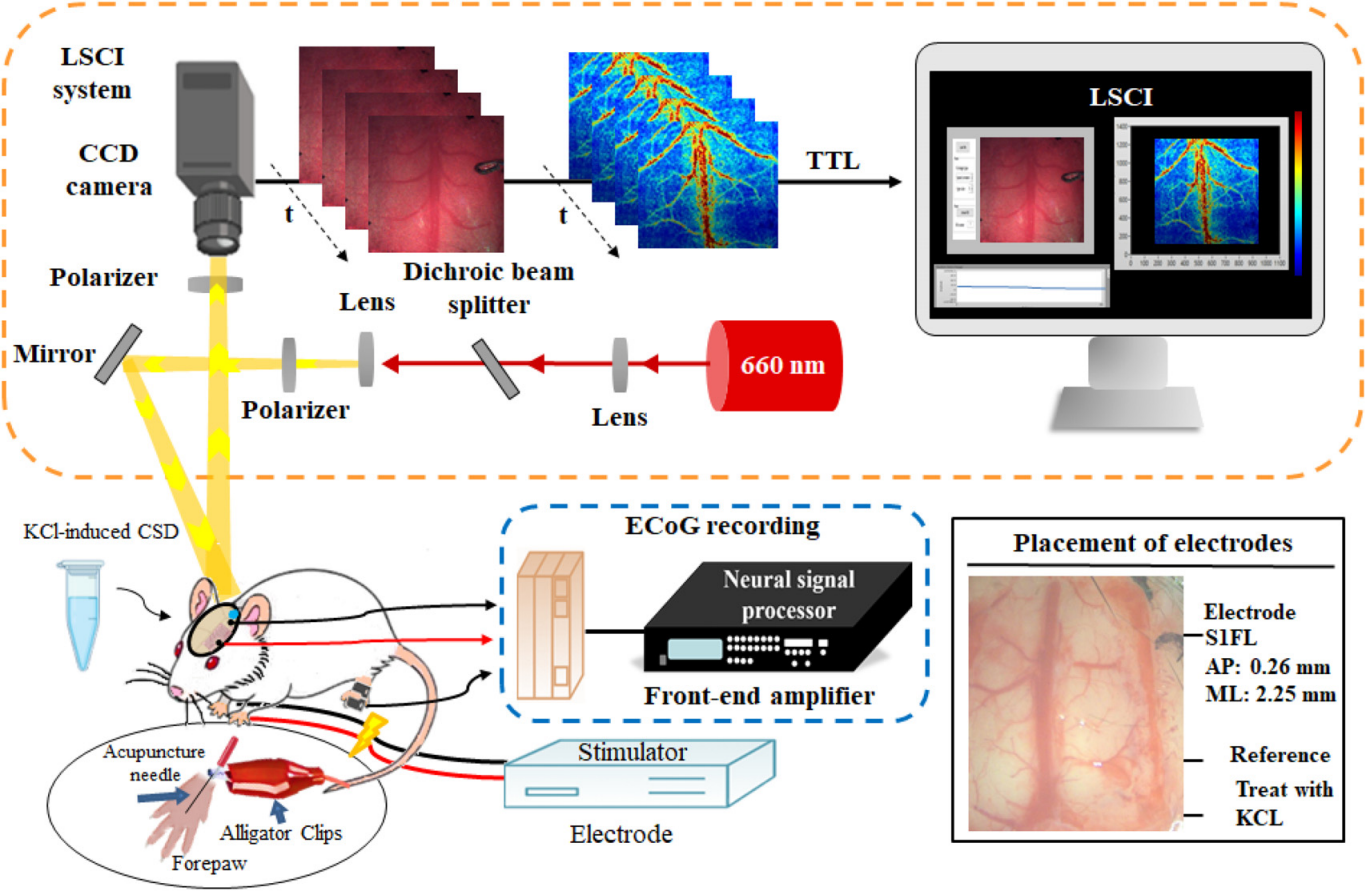
Schematic diagram of the ECoG-LSCI system and electrodes. ECoG signals and CBF were assessed via the ECoG-LSCI neurovascular imaging system. The system was used to detect changes in brain wave signals in mice produced in response to electrical stimulation of the front paws. Neural activity was measured through ECoG via screws implanted in the S1FL. Simultaneously, a 660-nm-wavelength laser was used to irradiate the cortex after craniotomy for LSCI. Images were captured with a computer via a CCD camera, and MATLAB was used to further analyze CBF. The figure shows the skull shape and spiral electrode locations, ECoG recording from the S1FL (AP = +0.26 mm and ML = +2.25 mm), the reference electrode location, and the KCl administration site (the upper right of the reference electrode is shown at the bottom right).

In this study, we developed an analytical strategy combining ECoG and LSCI, as shown in Fig. 9, to examine the effects of T1DM on cerebral neurovascular function. ECoG-LSCI allows simultaneous measurement of both rCBF and neuronal activity. To illuminate the region of interest, a 660-nm laser module (100 mW; RM-CW04-100, Unice E-O Service Inc., Taoyuan, Taiwan) was used. This laser emits light, and this light is then directed through a linear polarizer. A linear polarizer was positioned in front of the charge-coupled device (CCD) image collection lens at a working distance of approximately 5 cm. The purpose of this polarizer was to eliminate any specular reflectance that might interfere with the image collection process. By eliminating specular reflectance, the resulting images were made clearer and more detailed, increasing the quality of the information obtained for analysis. To properly illuminate the exposed area of the cortex, the laser beam was expanded through a plano–convex lens (75 mm; LA1608-A, Thorlabs Inc. Newton, NJ, USA) to a size of approximately 40 × 30 mm. The illuminated area was imaged by a 16-bit CCD camera (4.65 × 4.65 μm pixels; DR2-08S2M/C-EX-CS, Point Gray Research Inc. in Richmond, BC, Canada) via an adjustable magnification lens (0.3–1×, maximum f/4.5) with a 2× extender. Laser speckle images (1032 × 776 pixels) were obtained at 25 fps with an exposure time of 10 ms.^28^

LSCI was performed with LabVIEW software (National Instruments, Austin, TX, USA), which was developed in-house and utilizes the LabVIEW development platform. The analysis was performed via MATLAB software (version R2018a, provided by MathWorks Inc., Natick, MA, USA). To enhance the visualization of blood flow in real time with high resolution, a graphics processing unit (GPU) was integrated into the LSCI data processing framework. GPU technology, developed by NVIDIA (Santa Clara, CA, USA), was specifically used to accelerate image processing through parallel computing, significantly improving the computing performance. In this study, we used a GeForce GTX 650 Ti GPU from NVIDIA.

The images (taken upon illumination with the 660-nm laser) were passed through an amplifier that had a gain of 2 and a bandpass filter ranging from 0.5 Hz to 7500 Hz. The images were then digitized at a sampling rate of 1 kHz and subsequently filtered through a lowpass filter with a cutoff frequency of 100 Hz.

### B. Animals

The animals were kept in a controlled environment on a 12-h dark/light cycle at a constant temperature and humidity. Food and water were provided *ad libitum*. A total of 24 male C57BL/6 mice weighing between 20 and 25 g (National Laboratory Animal Center, Taiwan) were utilized and divided equally into two groups, the control and STZ groups, with 12 mice in each group. The health of the mice and their movement, fur condition, body weight homeostasis, and consumption of food and water were monitored four times a week. Because diabetic mice consume a large amount of water, water and food were replenished regularly, and the cages were frequently cleaned. The body weights and blood glucose levels of the mice were measured weekly. All animal experiments were conducted in strict adherence to the guidelines established by the Institutional Animal Care and Use Committee (IACUC) of the National Health Research Institute (NHRI), Taiwan, under the protocol number NHRI-IACUC-111001-A. In this study, if a mouse exhibited rapid weight loss of more than 20%, which was observed after the induction of diabetes, it was humanely euthanized through cervical dislocation under deep anesthesia by isoflurane.

### C. Diabetes induction

**FIG. 10.**
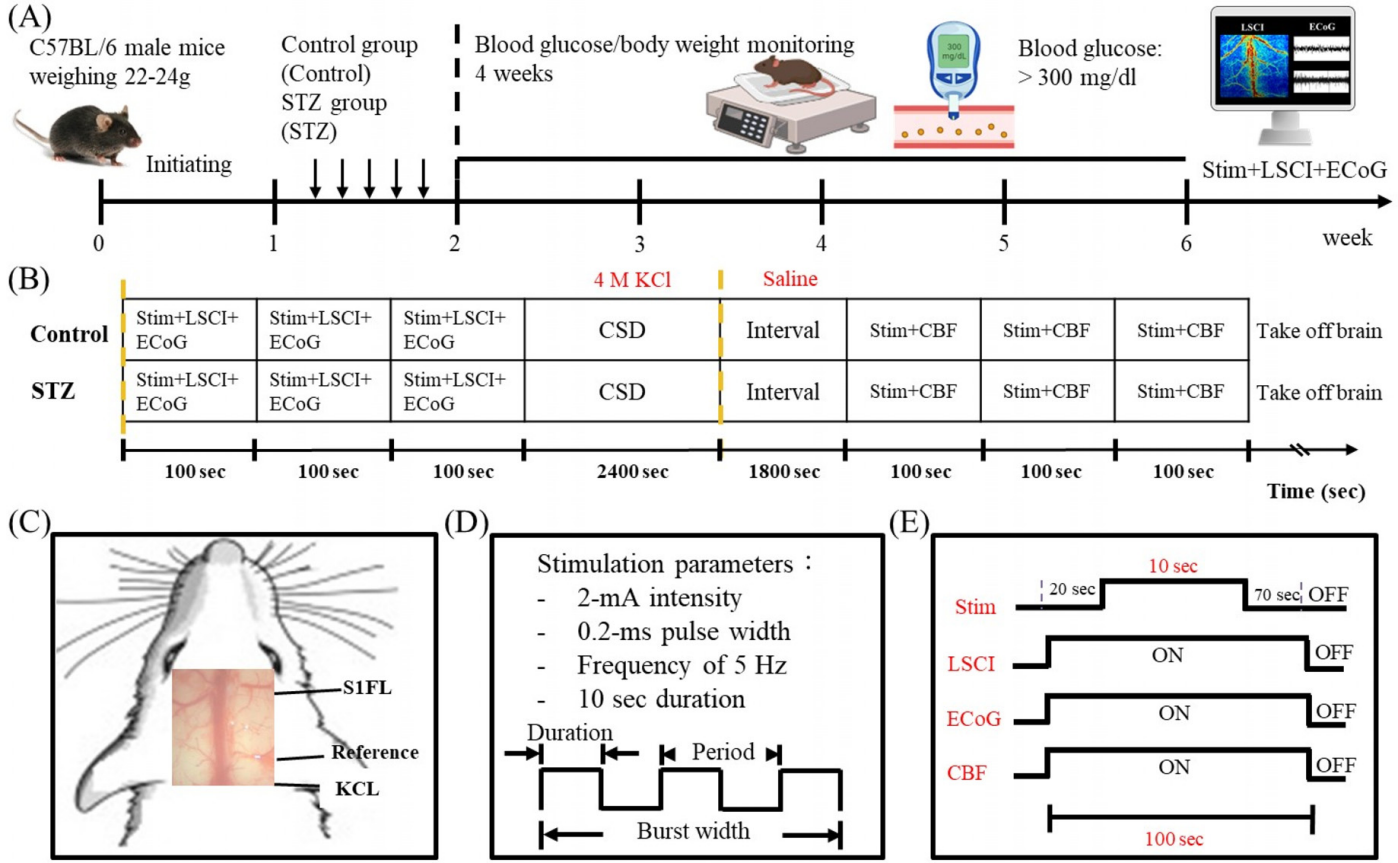
Experimental timeline of ECoG-LSCI after peripheral stimulation and construction of the STZ-induced diabetes animal model. (a) The mice were divided into two groups: the control group and the STZ group. The mice in the STZ group were treated with STZ (55 mg/kg) for five consecutive days, and the blood glucose levels and body weights of all the mice were monitored twice a week until day 28. Diabetic mice (defined as those with blood glucose levels ≥300 mg/dL) were identified 28 days after STZ administration. (b) ECoG-LSCI was used to measure signals in the S1FL after electrical stimulation. CSD was induced with 4 M KCl, and CSD and the change in CBF were then monitored for 40 min. (c) The location of the S1FL was determined by exposing the skull and drilling three holes: one for the ECoG electrode, one for the reference electrode, and one for KCl delivery. (d) Electrical signals in the S1FL were measured following 10 s of electrical stimulation at 2 mA. (e) ECoG-LSCI was used to monitor activity in the S1FL during three 10 s electrical stimulations (2 mA) separated by 100 s.

Diabetic mouse models were generated by the administration of STZ, an agent that is highly selective for and toxic to pancreatic β cells and induces complete β-cell necrosis within 48 h, specifically when it is administered at a single high dose and in diabetes models. The administration of multiple low doses of STZ only partially damages islets, triggering an inflammatory process that leads to further loss of β-cell activity and ultimately to insulin deficiency and hyperglycemia. Mouse diabetes models have obvious advantages, including small animal size, short induction period, ease of induction, and high cost-effectiveness. Diabetes induced by multiple low doses of STZ in mice may be more similar to human T1DM than hyperglycemia induced by a single high dose of STZ; moreover, male mice are more sensitive than female mice to multiple doses of STZ, and C57BL/6 mice are reliably sensitive to STZ. The STZ was stored at −20 °C to avoid degradation and was covered with an aluminum foil to protect it from light. STZ is unstable in solution, even at acidic pH values, so it was mixed with citrate buffer before injection. The STZ solution was freshly prepared and injected within 5 min of dissolution, as it can disintegrate in citrate buffer within 15–20 min, and to reduce variability, it was within 5 min of preparation. STZ was dissolved in citrate buffer (pH 4.5) and administered to mice intraperitoneally (i.p.) at a dose of 55 mg/kg body weight for five consecutive days.^5^ The control mice were treated with citrate buffer only. The blood glucose level was determined via an Accu-Chek glucose meter (Roche, Germany). To ensure the consistency of the test analysis values, the accuracy of each batch of products was confirmed. According to the original manufacturer's recommendations, the blood glucose meter was calibrated after replacement of the test strips. Mice with random blood glucose values ≥ 300 mg/dL were included in the STZ group [Fig. 10(a)].

### D. Animal preparation and surgery

The mice were subjected to craniotomy to create a window for ECoG-LSCI. The mice were anesthetized with 2% isoflurane (manufactured by Panion & BF Biotech Inc., Taoyuan, Taiwan) in oxygen [Fig. 10(b)]. A surgical drill was used to create a window ±2.0 mm anteroposterior (AP) and ±2.25 mm mediolateral (ML) to bregma. Two electrodes were implanted epidurally to record ECoG signals in the region of the primary somatosensory cortex (S1FL) corresponding to the forelimb at the following coordinates: AP = +0.26 mm and ML = +2.25 mm. Additionally, a reference electrode was placed +4 mm mediolateral to lambda, as depicted in Fig. 10(c).

### E. Peripheral electrical stimulation

The concentration of isoflurane was decreased to 1.5% before electrical stimulation. Two stainless steel needles were inserted into the left forelimb, one in the palm and the other in the proximal muscle of the forelimb. The limb was stimulated by applying rectangular pulse trains with a width of 0.2 ms at a rate of 5 Hz via a DS3 isolated current stimulator (Digitimer Ltd., Welwyn Garden City, Hertfordshire, UK). The maximum current amplitude was 2 mA. Each stimulation block consisted of 10 s of stimulation, which included 50 stimulation pulses separated by 10-s rest periods [as shown in Figs. 10(d) and 10(e)].

### F. Measuring blood flow via LSCI following electrical stimulation

The mice were anesthetized with isoflurane, and a midline incision was then made in the skin covering the skull. To measure CBF in mice, a highly sophisticated system known as the RFLSI Pro+ laser speckle system was used. This system, which was manufactured by RWD Life Science Co., Ltd. (in China), captured blood flow images with a resolution of 2048 × 2048. These images were then analyzed to identify specific ROIs. The ROIs were carefully selected on the basis of the anatomical features and functional requirements of the brain. Once the ROIs were identified, the system calculated the mean blood flow index for these regions in real time.

### G. KCl-induced CSD

Following SSEP measurements, CSD was induced with KCl [as illustrated in Fig. 10(b)]. To determine the CSD velocity, two points that were 2 mm apart were marked next to the imaging window via a stereotaxic system. A hole was drilled in the lower right corner during craniotomy to administer KCl for CSD induction, as shown in Fig. 10(c). After KCl was added, CSD-related events were recorded for 40 min. LSCI was used to determine the number of CSD-related events, the time point at which they occurred, and the CSD speed. A circular ROI was selected, and MATLAB was used to calculate the rCBF over time. The graph that was generated was used to determine the number of CSD events and the time points at which they occurred. To calculate the CSD speed, the distance between two ROIs was divided by the time difference in peaks between the two ROIs (X mm/Δt min). MATLAB software was used to calculate the CSD speed on the basis of the distance between the two points, the CSD time difference between the two ROIs, and the time difference between the peaks of the illuminated spots of the cortex. The CSD wave peak moved in the direction of the arrow, from the black line t1 in ROI1 to the blue line t2 in ROI2. P1 (X, Y) and P2 (X, Y) were marked 2 mm apart on the left side of the skull when craniotomy was performed. The coordinates were entered into the formula $\sqrt{{\left(P1X-P2X\right)}^{2}+{\left(P2Y-P2Y\right)}^{2}}$ to calculate the distance C between the two points, and the two specific ROIs in the image were then identified (circled by the arrow showing the CSD peak direction) to obtain the distance Z. The actual distance between the two ROIs X (mm) was subsequently calculated (C:2 = Z:X), and this distance was divided by the time difference to obtain the CSD speed (mm/s), which was then converted to mm/min.

### H. Analysis of LSCI data

To quantify the rCBF changes in the cortical region at different time points during CSD, normalized rCBF (rCBF_N_) was calculated as follows:

$$r{\text{CBF}}_{N}\left(T_{n}\right)=\frac{R\left(T_{n}\right)}{R\left(T_{b}\right)},$$ (1)

where $R(T_{b})$ is the baseline corresponding to the mean value of the resting rCBF fluctuations before CSD and $R(T_{n})$ is the mean value of the resting rCBF fluctuations in the *n*th time window in the control and STZ groups.

The linear correlation of the resting rCBF fluctuations in the cortical region at each frequency was evaluated by a magnitude-squared coherence function given by the following equation:

$${{\text{Coh}}^{2}}_{AM}\left(f\right)=\frac{{\left|P_{AM}\left(f\right)\right|}^{2}}{P_{AA}\left(f\right)P_{MM}\left(f\right)},$$ (2)

where *A* and *M* represent the rCBF signals of the cortex, $P_{AM}(f)$ represents the cross-power spectral density of the cortex, and $P_{AA}(f)$ and $P_{MM}(f)$ represent the power spectral densities of the cortex. Note that the power spectral density was estimated via Welch's overlapped averaged periodogram method.

In this study, the two rCBF signals were coherent at the frequency band $\left(f\right)$ between 0.05 and 0.15 Hz, where the coherence values were greater than 0.5.^29^ Therefore, the phase of $P_{AM}(f)$ at frequencies between 0.05 and 0.15 Hz was used to indicate the relative lag between the coherent components, which was calculated to describe the temporal relationship of the rCBF between the cortical regions and is defined as follows:

$$\emptyset \left(f\right)={\text{tan}}^{-1}\left[\frac{Im\left(P_{AM}\left(f\right)\right)}{Re\left(P_{AM}\left(f\right)\right)}\right],$$ (3)

where a positive phase difference ( ∅(f)) means that the CBF in the cortex lags behind that in the cortical area when blood is perfused through another cortical area, whereas a negative ∅(f) indicates that the CBF in the cortex lags behind that in the cortical region as the blood is perfused through another cortical region.^20^ The time difference was then calculated via the following equation:

$$\Delta t(f)=\frac{\emptyset \left(f\right)}{f}.$$ (4)

### I. Analysis of recorded ECoG data

The changes in SSEP responses to forepaw electrical stimulation in the control and STZ groups were evaluated by offline analysis via MATLAB. SSEPs were recorded, and their amplitudes were averaged over 50 sweeps to determine the average SSEP over a 0.2-ms period following stimulation. The average SSEP amplitude was then divided into two commonly observed components, P1 (first peak voltage after stimulation) and N1 (minimum voltage). The variations in the evoked responses, including the changes in the amplitude and peak latency, were assessed on the basis of these SSEP components.

### J. Protein extraction

After KCl-induced CSD analysis, each animal was sacrificed. The brain was removed and placed on ice, and the two hemispheres were separated. The hippocampus and cortex were dissected from each hemisphere and stored at −80 °C. For protein extraction, hippocampal and cortical tissues were homogenized in RIPA buffer (Visual Protein, Taiwan) containing protease and phosphatase inhibitor cocktail tablets (Roche, Germany). The homogenates were then centrifuged at 13 000 rpm for 15 min at 4 °C. The protein concentrations in the supernatants were measured with a protein assay kit (Bio-Rad, USA), and the protein samples were then stored at −80 °C.

### K. Western blotting

The samples were thawed on ice and then denatured in sample buffer (0.0625 M Tris, 2% [v/v] glycerol, 5% [w/v] sodium dodecyl sulfate [SDS], 5% [v/v] β-mercaptoethanol, and 0.001% [w/v] bromophenol blue, pH 6.8) containing 1% (v/v) protease and phosphatase inhibitor cocktail at 95 °C for 10 min. The samples (20 μg) were separated by gel electrophoresis on a 10% SDS–polyacrylamide gel at 60 V for 3 h and then transferred onto PVDF membranes via wet transfer (30 V at 4 °C overnight).

The membranes were blocked with 5% (w/v) nonfat milk in Tris-buffered saline (TBS) for 1 h at room temperature and then incubated for 2 h with antibodies at room temperature. The primary antibodies were diluted 1:1000 in blocking buffer. The primary antibodies used included anti-BDNF (ab108319; Abcam, UK), anti-NeuN (ab177487; Abcam, UK), anti-ADAM10 (ab124695; Abcam, UK), anti-BACE1 (ab183612; Abcam, UK), anti-P-AKT (S473) (Cat# 4060; Cell Signaling Technology, Danvers, MA, USA), anti-P-GSK3-β (Cat# 5558; Cell Signaling Technology, Danvers, MA, USA), and anti-actin (sc-47778; Santa Cruz, CA, USA) antibodies. The membranes were incubated for 2 h with peroxidase-conjugated anti-rabbit (ab6721; Abcam, UK) or anti-mouse (ab6789; Abcam, UK) IgG secondary antibodies, after which the bands were visualized via enhanced chemiluminescence (ECL) substrate (Bio-Rad, Hercules, CA, USA).

### L. Statistical analysis

Unpaired *t* tests were used to conduct column analyses of the experimental parameters for examination of the SSEP and CSD data. Multiple *t* tests were employed for grouped analyses (one per row) of the western blot data. All the statistical analyses were carried out with GraphPad Prism 8.0.1 (GraphPad Software, San Diego, CA, USA). The significance threshold was *p* < 0.05.

## SUPPLEMENTARY MATERIAL

See the supplementary material for the following: supplementary 1. video of dynamic changes in blood flow in the control group and supplementary 2. video of dynamic changes in blood flow in the STZ group.

## Data Availability

The data that support the findings of this study are available from the corresponding author upon reasonable request.
